# Supplementary material for: Chaperone mediated detection of small molecule target binding in cells
Source: Nat Commun. 2020 Jan 23;11:465. doi: 10.1038/s41467-019-14033-0 (PMC6978363; doi:10.1038/s41467-019-14033-0)
Supplement: Supplementary file 1 — Supplementary Information [file 41467_2019_14033_MOESM1_ESM.pdf]

A

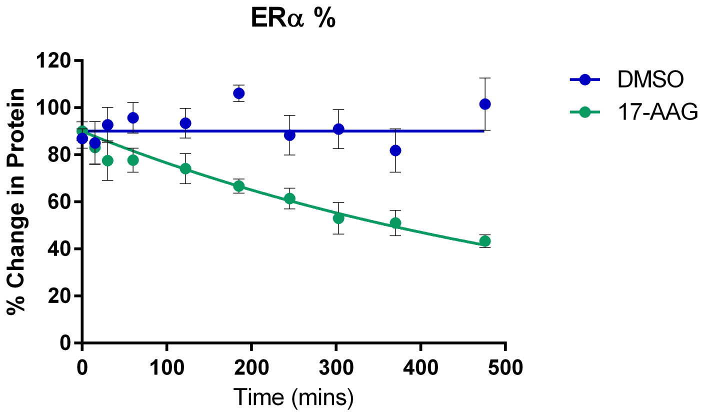

B

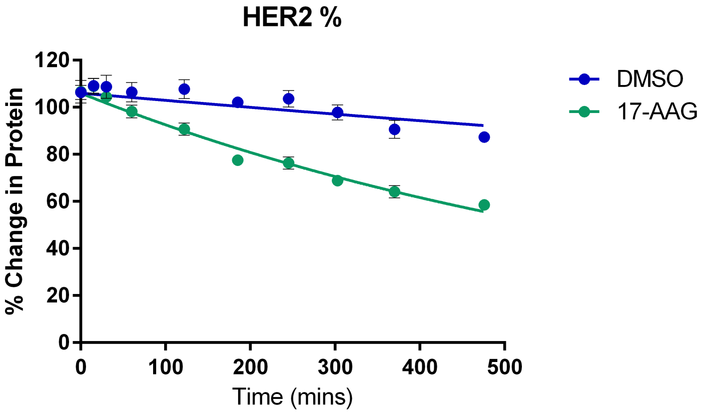

Supplementary Figure 1. Time course of destabilization of ERα and HER2 by 17-AAG.

A) The change in mean ERα protein per cell (MCF-7) was calculated for a time range using a single dose of 1 μM 17-AAG compared to DMSO control (half-life = 428 minutes). B) The change in mean HER2 protein per cell (MCF-7 neoHER2) was calculated for a time range using a single dose of 1 μM 17-AAG compared to DMSO control (half-life > 500 minutes). Data represent mean +/- standard deviation (n=6 to 8). Source data are provided as a Source Data file.

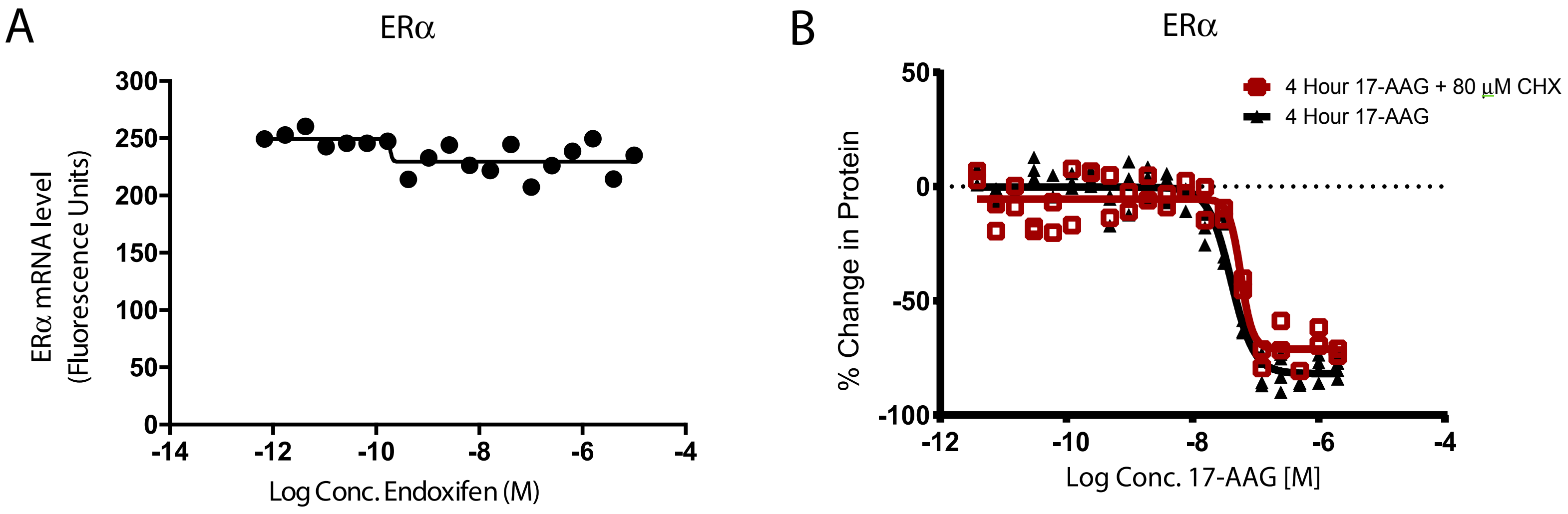

Supplementary Figure 2. Effects of Endoxifen on *ESR1* mRNA and cycloheximide on 17-AAG induced protein destabilization of ERα.

A) The change in *ESR1* mRNA levels measured (using the viewRNA branched-DNA (bDNA) based fluorescence in situ hybridization method; ThermoFisher Scientific) for a range of Endoxifen concentrations confirming that the effect of Endoxifen on ERα was not due to an indirect effect on *ESR1* mRNA levels. Individual data points are represented. B) The change in mean ERα protein per cell (MCF-7) was calculated for a range of 17-AAG concentrations with or without the addition of 80 μM cycloheximide, a protein synthesis inhibitor, demonstrating that the 17-AAG induced decrease in ERα is independent of *de novo* protein synthesis. Individual data points are represented (n=2 to 3). Source data are provided as a Source Data file.

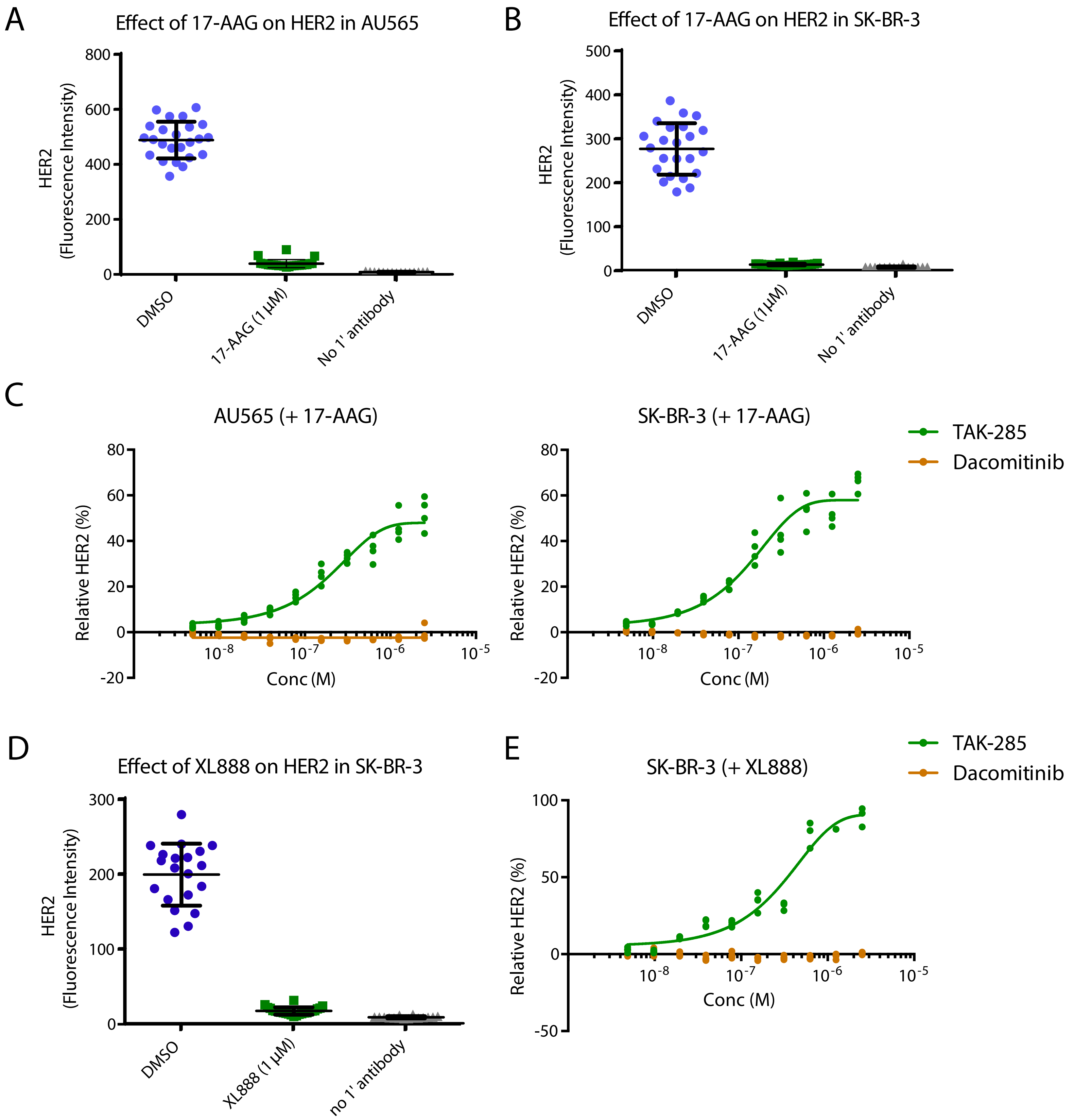

A
Effect of 17-AAG on HER2 in AU565
HER2
(Fluorescence Intensity)
800
600
400
200
0
DMSO
17-AAG (1 µM)
No 1' antibody
B
Effect of 17-AAG on HER2 in SK-BR-3
HER2
(Fluorescence Intensity)
500
400
300
200
100
0
DMSO
17-AAG (1 µM)
No 1' antibody
C
AU565 (+ 17-AAG)
SK-BR-3 (+ 17-AAG)
TAK-285
Dacomitinib
Relative HER2 (%)
80
60
40
20
0
-20
10-8
10-7
10-6
10-5
Conc (M)
D
Effect of XL888 on HER2 in SK-BR-3
HER2
(Fluorescence Intensity)
300
200
100
0
DMSO
XL888 (1 µM)
no 1' antibody
E
SK-BR-3 (+ XL888)
TAK-285
Dacomitinib
Relative HER2 (%)
100
50
0
-50
10-8
10-7
10-6
10-5
Conc (M)

Supplementary Figure 3. Effects of TAK-285 and 17-AAG on HER2 in AU565 and SK-BR-3 cell lines.

A-B) 17-AAG (1 μM) reduces HER2 protein levels in AU565 (A) and SK-BR-3 (B) cell lines based on quantitative immunofluorescence intensities. Data are mean +/- standard deviation (n=16 to 24). C) TAK-285 pre-treatment over a range of concentrations stabilizes HER2 partially protecting it from 17-AAG effects in both AU565 and SK-BR-3. This effect was not seen for the EGFR inhibitor Dacomitinib. Individual data points are shown (n=4). D) HSP90 inhibitor XL888 (1 μM) reduces HER2 protein levels in SK-BR-3 cell line based on quantitative immunofluorescence intensities. Data are mean +/- standard deviation (n=16 to 24). E) TAK-285 pre-treatment over a range of concentrations stabilizes HER2 partially protecting it from XL888 effects in SK-BR-3. This effect was not seen for the EGFR inhibitor Dacomitinib. Individual data points are shown (n= 2 to 4). Relative HER2 protein levels are expressed as the percentage of HER2 present in the co-treatment condition relative to the corresponding concentration of inhibitor only. Source data are provided as a Source Data file.

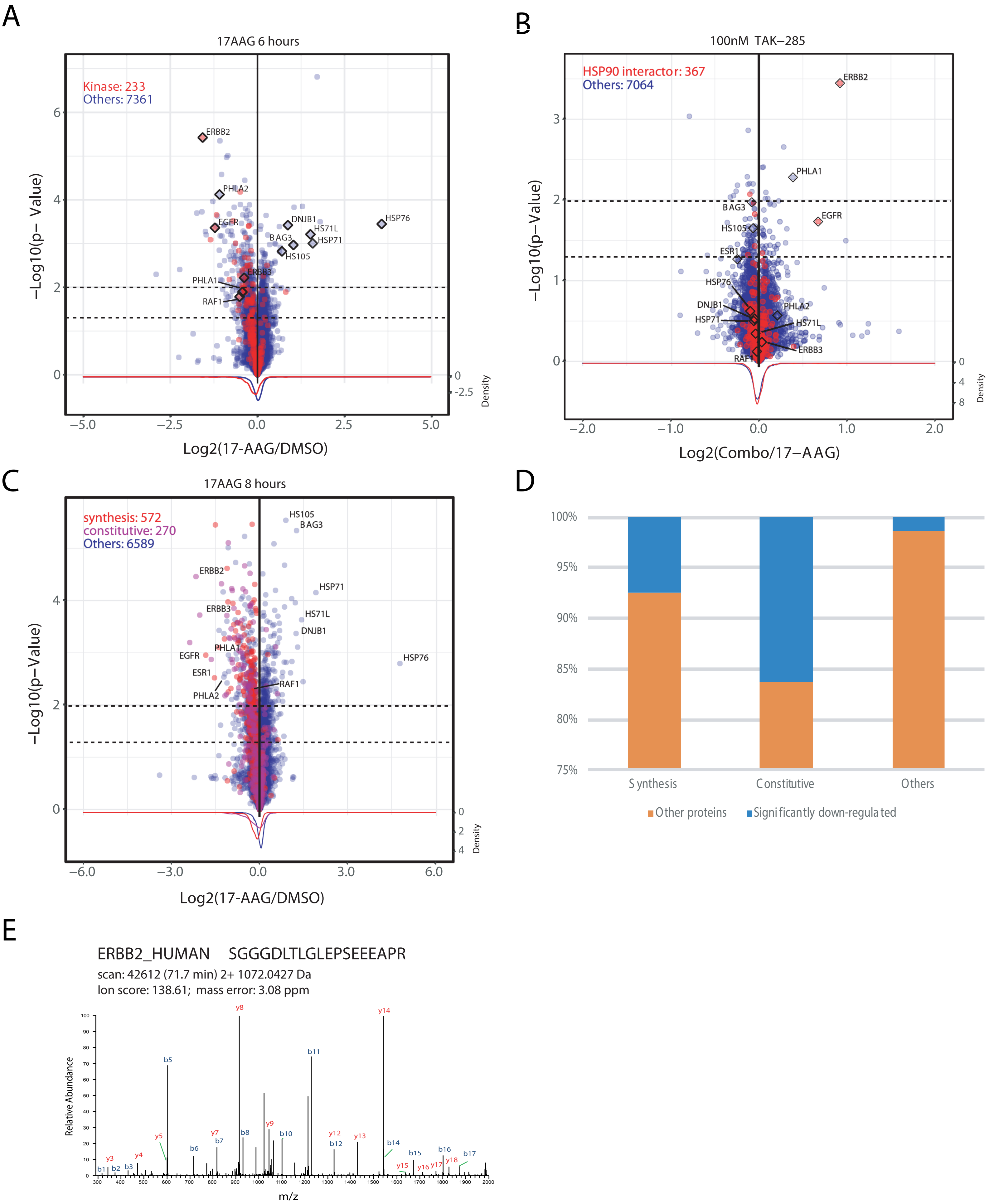

A
17AAG 6 hours
Kinase: 233
Others: 7361
ERBB2
PHLA2
EGFR
DNJB1
HSP76
HS71L
HSP71
BAG3
HS105
ERBB3
PHLA1
RAF1
−Log10(p− Value)
Log2(17-AAG/DMSO)
Density
B
100nM TAK−285
HSP90 interactor: 367
Others: 7064
ERBB2
PHLA1
EGFR
BAG3
HS105
ESR1
HSP76
DNJB1
HSP71
PHLA2
HS71L
ERBB3
RAF1
−Log10(p−Value)
Log2(Combo/17−AAG)
Density
C
17AAG 8 hours
synthesis: 572
constitutive: 270
Others: 6589
HS105
BAG3
ERBB2
HSP71
ERBB3
HS71L
DNJB1
PHLA1
EGFR
HSP76
ESR1
RAF1
PHLA2
−Log10(p−Value)
Log2(17-AAG/DMSO)
Density
D
Synthesis
Constitutive
Others
Other proteins
Significantly down-regulated
E
ERBB2_HUMAN SGGGDLTLGLEPSEEEAPR
scan: 42612 (71.7 min) 2+ 1072.0427 Da
Ion score: 138.61; mass error: 3.08 ppm
Relative Abundance
m/z

Supplementary Figure 4. Mass spectrometry-based global proteomics profiling after TAK-285 treatment.

A) Volcano plot depicting the fold change in protein abundance in MCF7 neoHER2 cells treated with 17-AAG (1 μM; 6 hours) relative to DMSO only control samples. The horizontal axis indicates the relative change in protein caused by 17-AAG treatment; Log2(17-AAG/DMSO) is the log2 transformed ratio of the protein levels in the 17-AAG treated samples and the DMSO treated samples. The vertical axis represents the statistical significance; -log10 transformed p-value (dotted lines indicate p=0.05 and p=0.01). Each circle represents an individual protein with proteins annotated as kinase (red) or others (blue) and density curves for the two protein groups are shown at the bottom and their y-axis is shown on the right. Annotated proteins and alternative gene names include: ERBB2 (HER2), PHLA1 (PHLDA1), PHLA2 (PHLDA2), BAG3, HS105, ESR1, HSP76 (HSPA6), DNJB1, HSP71 (HSPA1A), HS71L (HSPA1L), RAF1 (CRAF), ERBB3, EGFR. B) Volcano plot depicting the fold change in protein abundance in MCF7 neoHER2 cells treated with a combination of 17-AAG (1 μM) and TAK-285 (100 nM) relative to 17-AAG only samples (combo/17-AAG) for 8 hours. Horizontal and vertical axes are similar to those described for A). Proteins are annotated as HSP90 interactors (red) or others (blue) and density curves for the two protein groups are shown at the bottom and their y-axis is shown on the right. C) Volcano plot depicting the fold change in protein abundance in MCF7 neoHER2 cells treated with 17-AAG (1 μM; 8 hours) relative to DMSO only control samples. The axes are as defined for A). Proteins are annotated by HSP90 dependence: HSP90 dependence during protein synthesis (red), constitutive HSP90 dependence (magenta), or others (blue) and density curves for the different protein groups are shown at the bottom and their y-axis is shown on the right. D) Fractions of proteins significantly down-regulated (blue) in each

HSP90 dependency group, i.e. those proteins annotated as being dependent on HSP90 during synthesis, or constitutively, or others (unknown). E) Identification of ERBB2 protein: An MS/MS spectrum matching to one of the unique ERBB2 peptides. Source data are provided as a Source Data file.

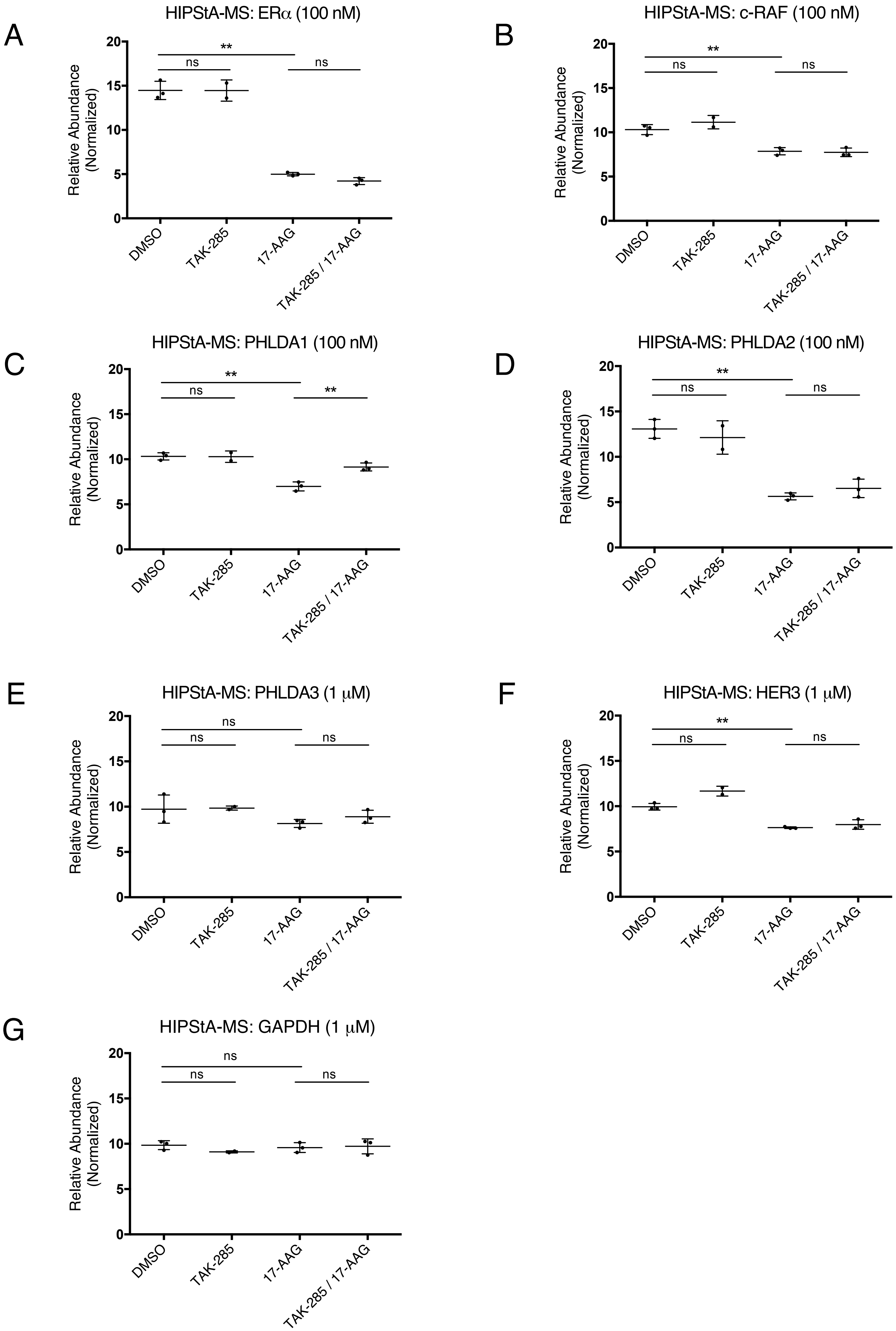
A
HIPStA-MS: ERα (100 nM)
B
HIPStA-MS: c-RAF (100 nM)
C
HIPStA-MS: PHLDA1 (100 nM)
D
HIPStA-MS: PHLDA2 (100 nM)
E
HIPStA-MS: PHLDA3 (1 μM)
F
HIPStA-MS: HER3 (1 μM)
G
HIPStA-MS: GAPDH (1 μM)
Relative Abundance (Normalized)
0
5
10
15
20
**
ns
DMSO
TAK-285
17-AAG
TAK-285 / 17-AAG

Supplementary Figure 5. Quantitative mass spectrometry data for a subset of proteins from various TAK-285 HIPStA global proteomics profiling studies.

The graphs show the relative protein abundance in MCF-7 neoHer2 cells for the four different treatment groups (DMSO, TAK-285, 17-AAG and the combination of TAK-285 and 17-AAG, for A) ERα (100 nM TAK-285), B) c-RAF (100 nM TAK-285), C) PHLDA1 (100 nM TAK-285), D) PHLDA2 (100 nM TAK-285), E) PHLDA3 (1 μM TAK-285), F) HER3 (1 μM TAK-285), and G) GAPDH (1 μM TAK-285). Individual data points are calculated as the percentage of sum total TMT signal for the protein (across all treatment groups), and shown with mean and standard deviation indicated. The statistical significance of the relative abundance of each protein in the different samples were determined using two-tailed student's t-tests for: TAK-285 vs. DMSO, 17-AAG vs. DMSO, and TAK-285 + 17-AAG vs. 17-AAG; $p<0.05$ (*), $p<0.01$ (**), $p<0.001$ (***), $p<0.0001$ (****). ERBB3 (HER3), a family member of ERBB2, was destabilized by 17-AAG in the global proteome profiling studies, but this destabilization was not rescued by pre-incubation with TAK-285, suggesting that TAK-285 does not bind ERBB3 (HER3). Similarly, the nuclear hormone receptor ERα (ESR1) and c-RAF proteins were also observed to be destabilized by 17-AAG, but this destabilization was not prevented by prior incubation with TAK-285. Source data are provided as a Source Data file.

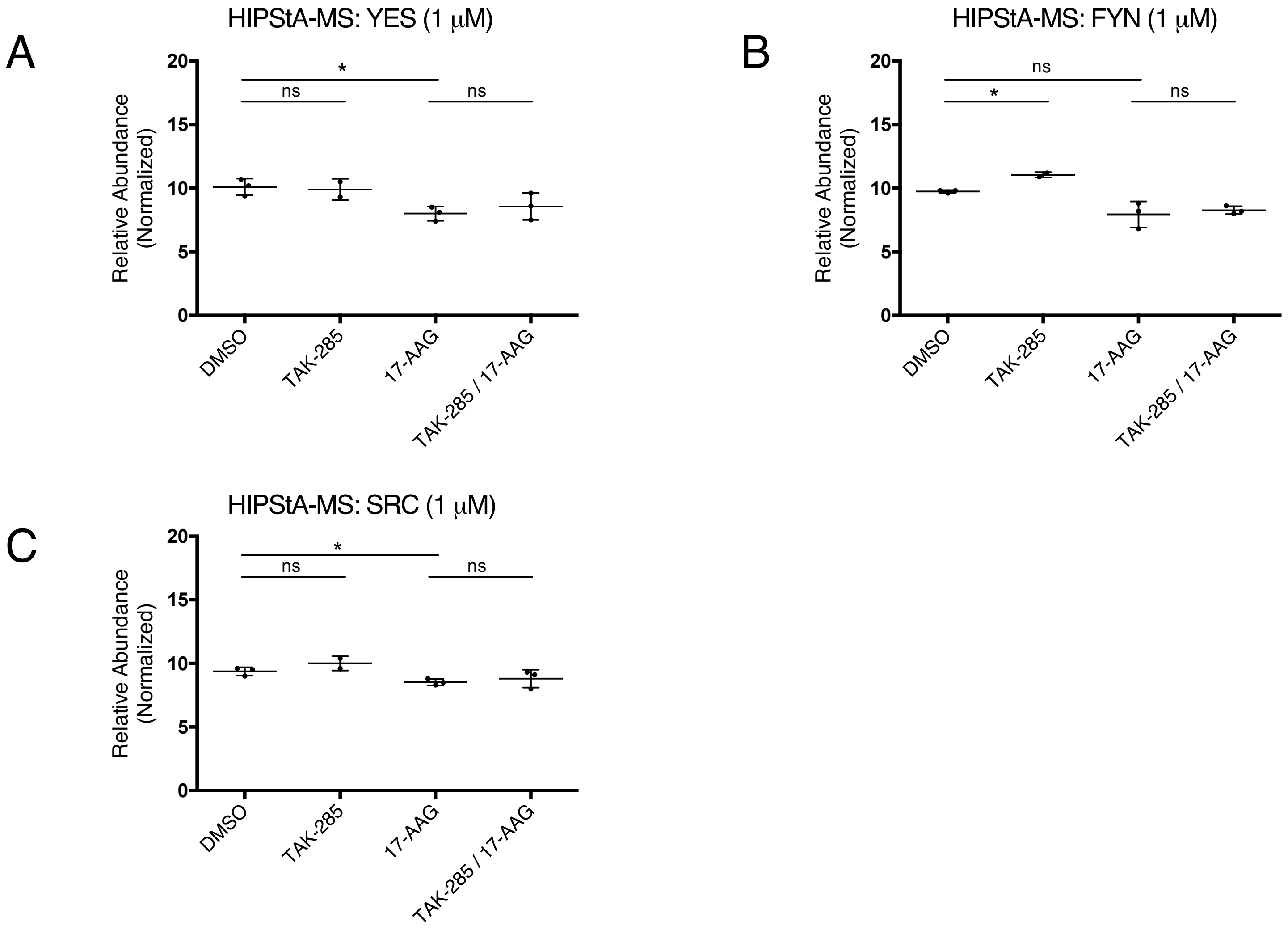

Supplementary Figure 6. Quantitative mass spectrometry data for a subset of Src family kinases from various TAK-285 HIPStA global proteomics profiling studies.

The graphs show the relative protein abundance in the four different treatment groups for A) YES (1 μM TAK-285), B) FYN (1 μM TAK-285), and C) SRC (1 μM TAK-285). Individual data points are calculated as the percentage of sum total TMT signal for the protein (across all treatment groups), and shown with mean and standard deviation indicated. The statistical significance of the relative abundance of each protein in the different samples were determined using two-tailed student's t-tests for: TAK-285 vs. DMSO, 17-AAG vs. DMSO, and TAK-285 + 17-AAG vs. 17-AAG ; $p<0.05$ (*), $p<0.01$ (**), $p<0.001$ (***), $p<0.0001$ (****). Source data are provided as a Source Data file.

A

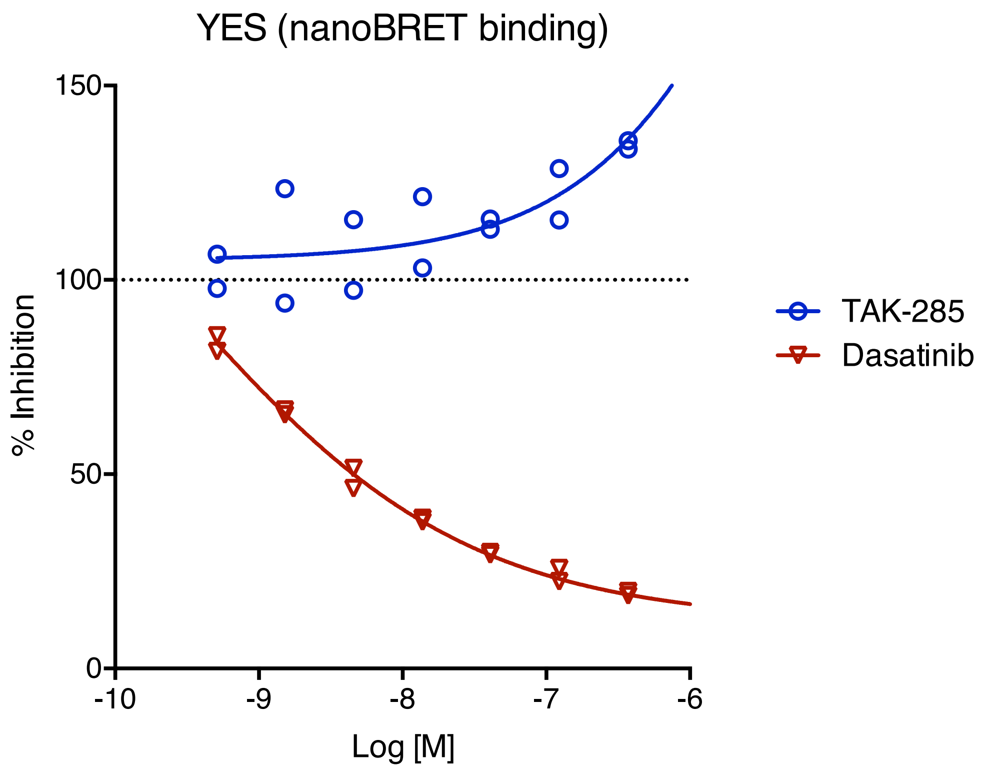

Supplementary Figure 7. Assessing TAK-285 binding to Yes kinase using a nanoBRET assay.

A) Cell-based NanoBRET assay to detect whether TAK-285 binds to the Yes tyrosine kinase, compared to a positive control, dasatinib. Data represent individual duplicate data points. An increase in fluorescence at higher concentrations of TAK-285 may reflect fluorescence artifacts, but it remains clear that there is no binding to Yes in cells in this context. Source data are provided as a Source Data file.

A

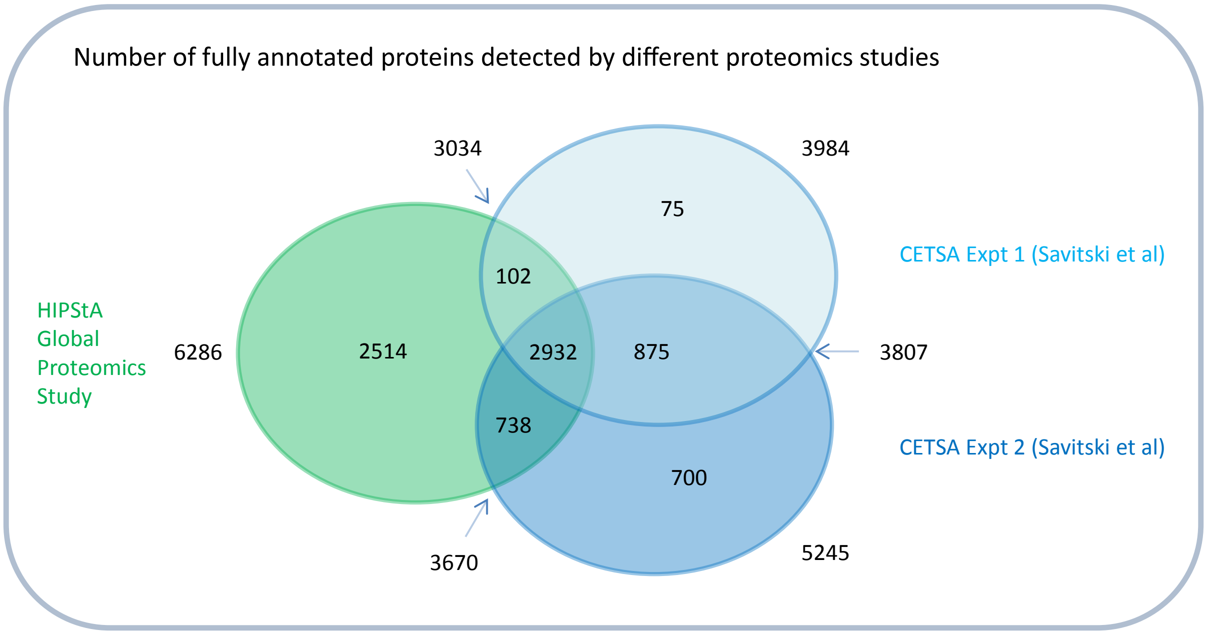

Number of fully annotated proteins detected by different proteomics studies
3034
3984
75
CETSA Expt 1 (Savitski et al)
102
HIPStA
Global
Proteomics
Study
6286
2514
2932
875
3807
738
CETSA Expt 2 (Savitski et al)
700
3670
5245

B

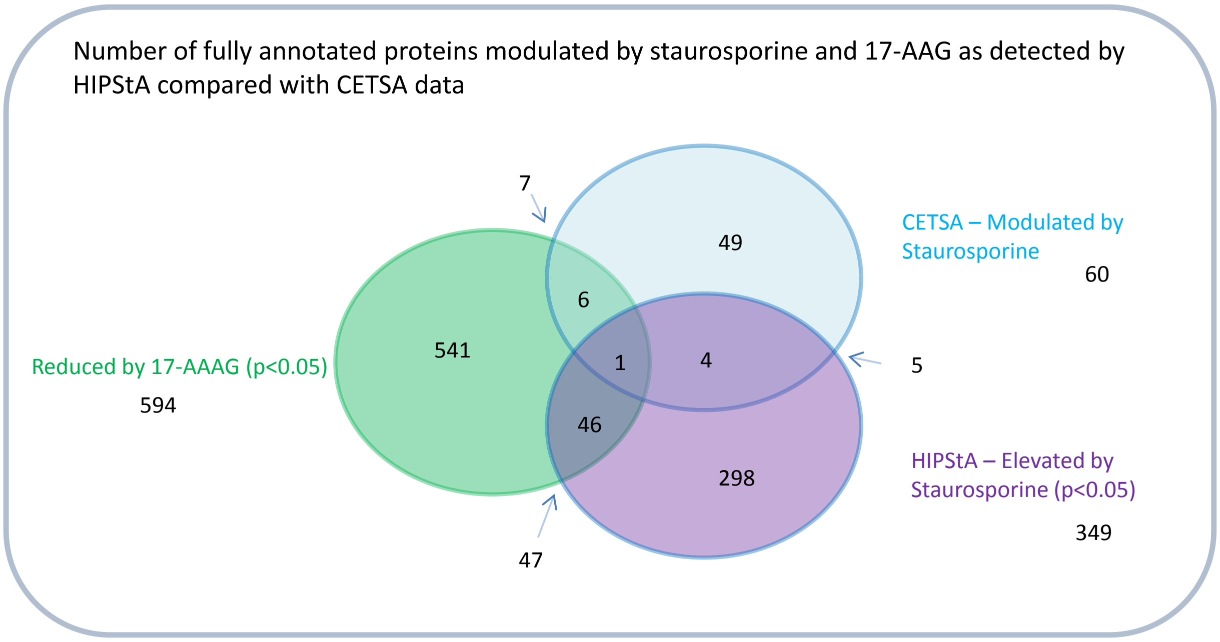

Number of fully annotated proteins modulated by staurosporine and 17-AAG as detected by HIPStA compared with CETSA data
7
49
CETSA – Modulated by
Staurosporine
60
6
541
Reduced by 17-AAAG (p<0.05)
594
1
4
5
46
HIPStA – Elevated by
Staurosporine (p<0.05)
298
349
47

C

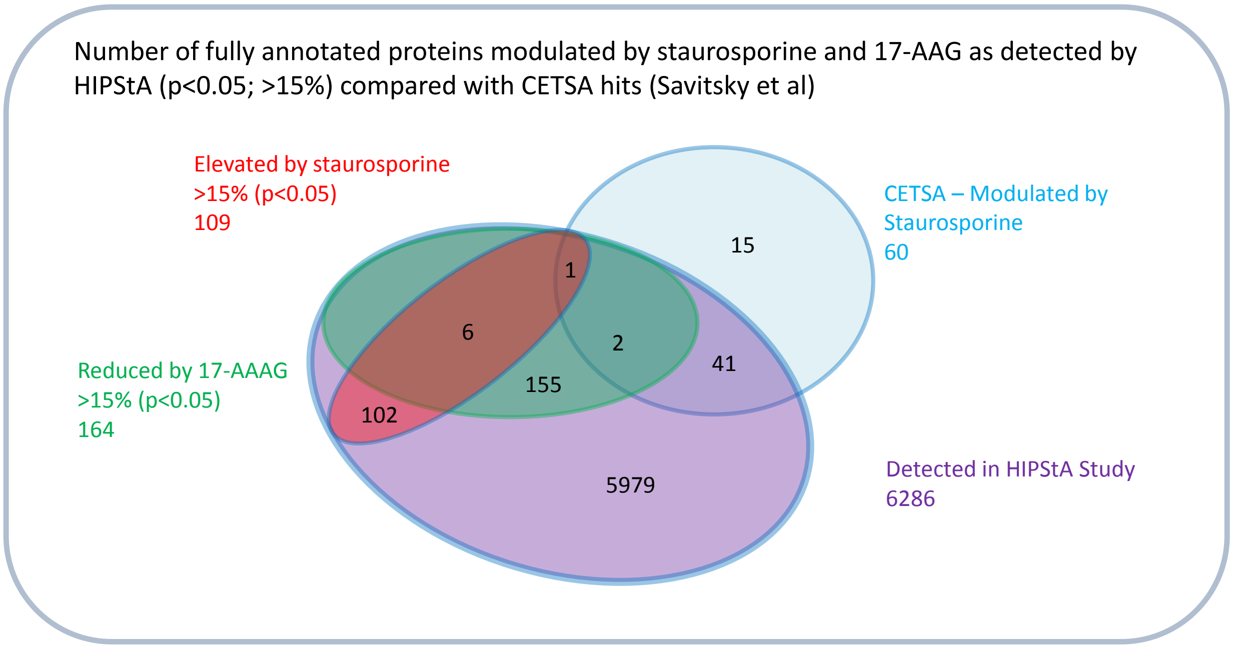

Number of fully annotated proteins modulated by staurosporine and 17-AAG as detected by HIPStA (p<0.05; >15%) compared with CETSA hits (Savitsky et al)
Elevated by staurosporine
>15% (p<0.05)
109
CETSA – Modulated by
Staurosporine
60
15
1
6
2
Reduced by 17-AAAG
>15% (p<0.05)
164
155
41
102
Detected in HIPStA Study
6286
5979

Supplementary Figure 8. Comparison between HIPStA and CETSA studies of staurosporine: Protein detection and hit overlap.

A HIPStA global proteomics profiling study of the effects of staurosporine (1μM) in MCF-7 neoHer2 cells. Venn diagrams depicting of the overlap between the HIPStA global proteomics profiling study of the effect of staurosporine on protein stability and published CETSA studies of the effect of staurosporine on protein stability. The tabulated data from Savitski *et al*[39]were combined with the HIPStA global proteomics profiling data depicted in Supplemental Figure 8 using gene names to match the data sets. Only data for fully annotated genes were used for the data comparison. A) The number of fully annotated proteins that were detected in the HIPStA global proteomics profiling are compared to 2 CETSA global proteomics profiling experiments. The HIPStA study protein set (6286 annotated proteins) overlapped with the 2 individual CETSA protein sets (3984 and 5245 annotated proteins) by 48.3% (3034/6286) and 58.4% (3670/6286); and overlapped by 46.6% (2932/6286) with the overlapping CETSA protein sets. B) The number of fully annotated proteins that were modulated by staurosporine
($p<0.05$) and reduced by 17-AAG ($p<0.05$), as detected by HIPStA, are compared with the 60 proteins that were defined as staurosporine interactors by CETSA (CETSA 60 hit set). C) The fully annotated HIPStA study protein set (6286 proteins) is compared to the CETSA 60 hit set, and sub-divided into the number of proteins that were reduced by greater than 15% ($p<0.05$) by 17-AAG or elevated by staurosporine greater than 15% ($p<0.05$) in the HIPStA study. Within the CETSA 60 hit set, 43 were detected in the HIPStA study, with 3 of these being reduced by 17-AAG (>15%, $p<0.05$) and 1 (ADBRK1) also being stabilized by staurosporine (>15%, $p<0.05$). Using these criteria, the HIPStA study identified 6 other staurosporine modulated proteins, which were not detected in the CETSA study. Source data are provided as a Source Data file.

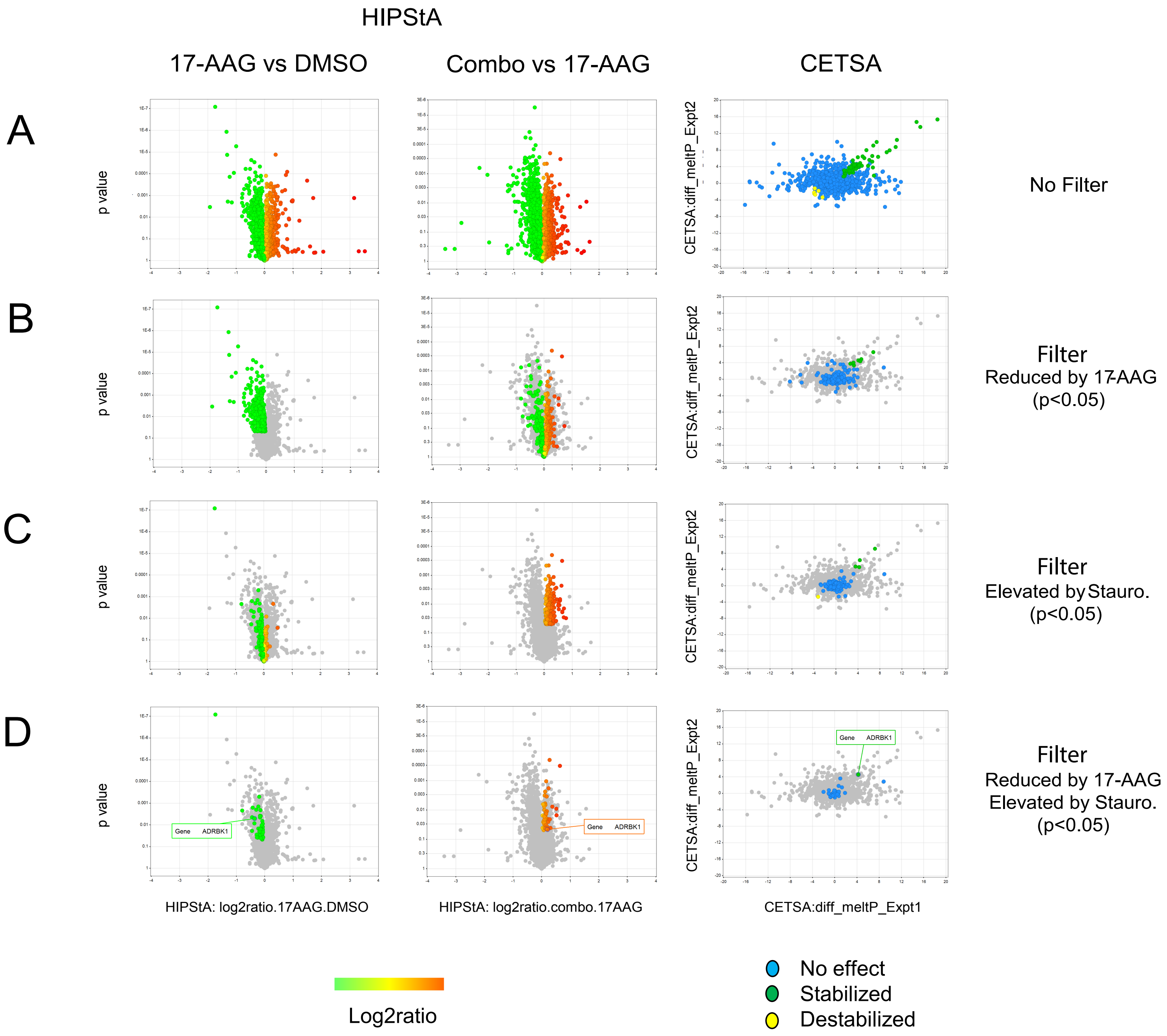
HIPStA
17-AAG vs DMSO
Combo vs 17-AAG
CETSA
A
B
C
D
p value
CETSA:diff_meltP_Expt2
No Filter
Filter
Reduced by 17-AAG
(p<0.05)
Filter
Elevated by Stauro.
(p<0.05)
Filter
Reduced by 17-AAG
Elevated by Stauro.
(p<0.05)
Gene ADRBK1
HIPStA: log2ratio.17AAG.DMSO
HIPStA: log2ratio.combo.17AAG
CETSA:diff_meltP_Expt1
Log2ratio
No effect
Stabilized
Destabilized

Supplementary Figure 9. Comparison between HIPStA and CETSA studies of staurosporine: Hit selection criteria.

A HIPStA global proteomics profiling study of the effects of staurosporine (1μM) in MCF-7 neoHer2 cells. The data for the overlapping protein set that was detected in both the HIPStA global proteomics staurosporine study and the overlapping CETSA protein sets[39] are compared (2932 proteins). The effect of each HIPStA hit selection filter is depicted in A-D. Volcano plots (left and center panels) show individual points depicting individual proteins in the HIPStA study. The left panels show the change in protein levels caused by 17-AAG (17-AAG vs DMSO). The center panels show the effect that pretreatment (combination) with staurosporine (Combo vs 17-AAG) had on these changes. The vertical axis indicates statistical significance (p value) and the horizontal axis indicates the log2ratio of the relative fold change. The color indicates the relative change in protein level with green indicating a relative decrease (log2ratio < 0) and orange indicating a relative increase (log2ratio > 0). The right panels show CETSA data (from Savitski *et al*[39]) for the same group of proteins, depicted as scatter plots with individual points depicting the CETSA differential change in melting point caused by staurosporine for each individual protein, measured in two experiments (horizontal and vertical axis). Proteins that demonstrated a consistent increase or decrease in differential melting point caused by staurosporine are depicted in green or yellow (respectively). Proteins that have been excluded by the various HIPStA exclusion filters in each data set are shown in grey. Source data are provided as a Source Data file.

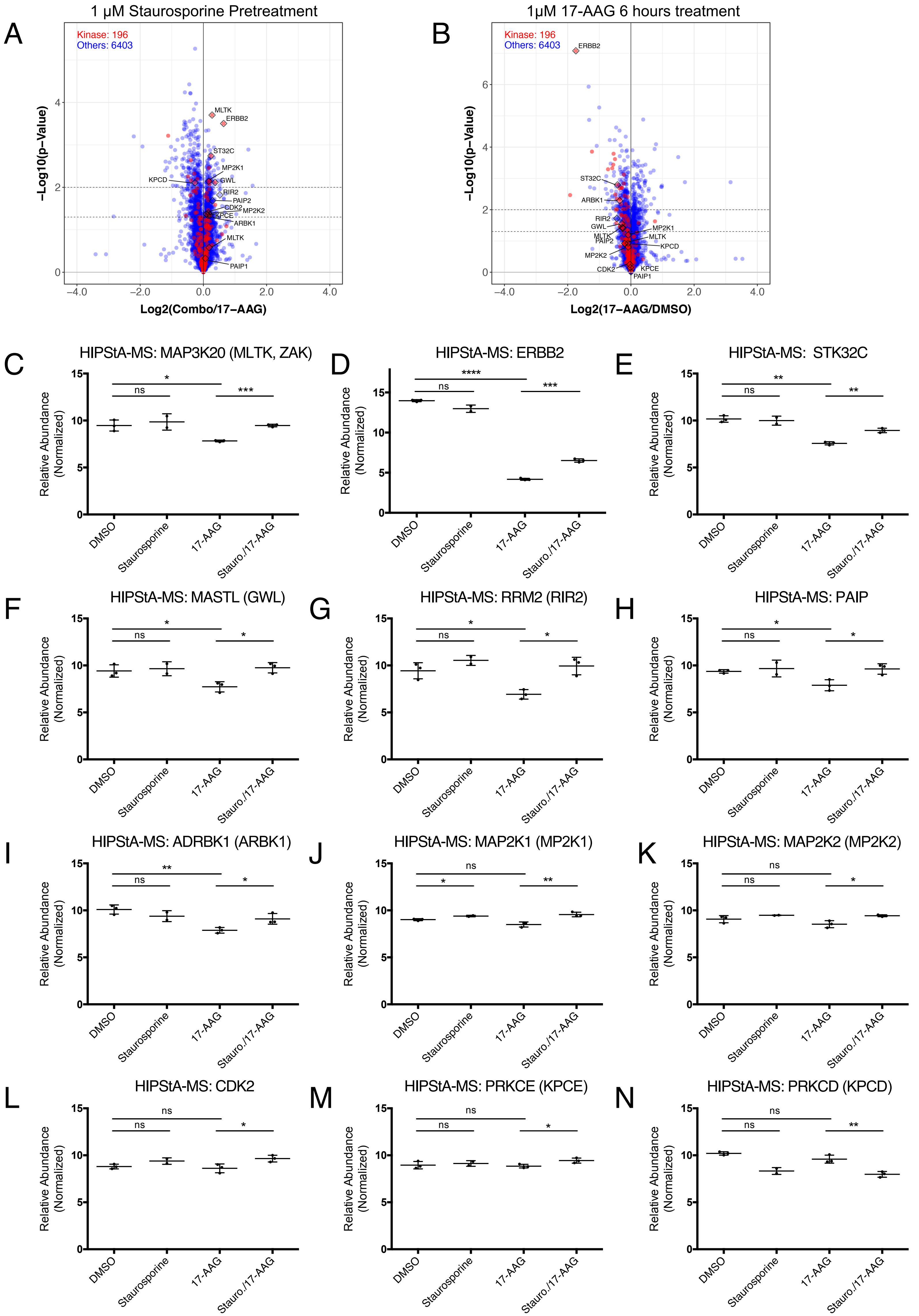

A
1 μM Staurosporine Pretreatment
Kinase: 196
Others: 6403
-Log10(p-Value)
Log2(Combo/17-AAG)
MLTK
ERBB2
ST32C
MP2K1
KPCD
GWL
RIR2
PAIP2
CDK2
MP2K2
KPCE
ARBK1
MLTK
PAIP1
B
1μM 17-AAG 6 hours treatment
Kinase: 196
Others: 6403
ERBB2
ST32C
ARBK1
RIR2
GWL
MLTK
PAIP2
MP2K1
MLTK
KPCD
MP2K2
CDK2
KPCE
PAIP1
-Log10(p-Value)
Log2(17-AAG/DMSO)
C
HIPStA-MS: MAP3K20 (MLTK, ZAK)
D
HIPStA-MS: ERBB2
E
HIPStA-MS: STK32C
F
HIPStA-MS: MASTL (GWL)
G
HIPStA-MS: RRM2 (RIR2)
H
HIPStA-MS: PAIP
I
HIPStA-MS: ADRBK1 (ARBK1)
J
HIPStA-MS: MAP2K1 (MP2K1)
K
HIPStA-MS: MAP2K2 (MP2K2)
L
HIPStA-MS: CDK2
M
HIPStA-MS: PRKCE (KPCE)
N
HIPStA-MS: PRKCD (KPCD)
Relative Abundance (Normalized)
DMSO
Staurosporine
17-AAG
Stauro./17-AAG

Supplementary Figure 10. HIPStA global proteomics profiling study examining the effect of the non-specific kinase inhibitor staurosporine.

A HIPStA global proteomics profiling study of the effects of staurosporine (1μM) in MCF-7 neoHer2 cells identified 6599 proteins, of which 6286 were fully annotated proteins and 196 were kinases. The method was similar to that defined in the schematic in Figure 4A except testing staurosporine (instead of TAK-285). A) Volcano plot of the relative change in protein levels caused by the pre-incubation with staurosporine (1 μM) prior to treatment with 17-AAG (1 μM), with relative changes in protein level shown on the horizontal axis as Log2(Combo/17-AAG), which is the log2 transformed ratio of the protein levels in the combination treated samples and the 17-AAG treated samples. The vertical axis shows the statistical significance: -log10 transformed p-value (-Log10(p-value); dotted lines indicate p=0.05 and p=0.01). B) Volcano plot of the relative changes in protein levels caused by 6 hour treatment with 1μM 17-AAG shown on the horizontal axis as log2(17-AAG/DMSO) versus the statistical significance of the change in protein level (-Log10(p-value) shown on the vertical axis. Each point represents an individual protein with kinases depicted in red and other proteins depicted in blue. The relative protein abundances across the different treatment groups for selected proteins are shown in: C) MAP3K20 (MLTK, ZAK); D) ERBB2 (HER2); E) STK32C; F) MASTL (GWL); G) RRM2 (RIR2); H) PAIP; I) ADRBK1 (ARBK1); J) MAP2K1 (MP2K1); K) MAP2K2 (MP2K2); L) CDK2; M) PRKCE (KPCE); N) PRKCD (KPCD). Individual data points are calculated as the percentage of sum total TMT signal for the individual protein (across all treatment groups), and shown with mean and standard deviation indicated. The statistical significance of the relative abundance of each protein in the different samples was determined using two-tailed student's t-tests; $p<0.05$ (*), $p<0.01$ (**), $p<0.001$ (***), $p<0.0001$ (****). Source data are provided as a Source Data file.

EGFR_HUMAN|P00533

Coverage: 36/1210 (3%)

1 MRPSGTAGAA LLALLAALCP ASRALEEKKV CQGTSNKLTQ LGTFEDHFLS
51 LQRMFNNCEV VLGNLEITYV QRNYDLSFLK TIQEVAGYVL IALNTVERIP
101 LENLQIIRGN MYYENSYALA VLSNYDANKT GLKELPMRNL QEILHGAVRF
151 SNNPALCNVE SIQWRDIVSS DFLSNMSMDF QNHLGSCQKC DPSCPNGSCW
201 GAGEENCQKL TKIICAQQCS GRCRGKSPSD CCHNQCAAGC TGPRESDCLV
251 CRKFRDEATC KDTCPPLMLY NPTTYQMDVN PEGKYSFGAT CVKKCPRNYV
301 VTDHGSCVRA CGADSYEMEE DGVRKCKKCE GPCRKVCNGI GIGEFKDSLS
351 INATNIKHFK NCTSISGDLH ILPVAFRGDS FTHTPPLDPQ ELDILKTVKE
401 ITGFLLIQAW PENRTDLHAF ENLEIIRGRT KQHGQFSLAV VSLNITSLGL
451 RSLKEISDGD VIISGNKNLC YANTINWKKL FGTSGQKTKI ISNRGENSCK
501 ATGQVCHALC SPEGCWGPEP RDCVSCRNVS RGRECVDKCN LLEGEPREFV
551 ENSECIQCHP ECLPQAMNIT CTGRGPDNCI QCAHYIDGPH CVKTCPAGVM
601 GENNTLVWKY ADAGHVCHLC HPNCTYGCTG PGLEGCPTNG PKIPSIATGM
651 VGALLLLLVV ALGIGLFMRR RHIVRKRTLR RLLQERELVE PLTPSGEAPN
701 QALLRILKET EFKKIKVLGS GAFGTVYKGL WIPEGEKVKI PVAIKELREA
751 TSPKANKEIL DEAYVMASVD NPHVCRLLGI CLTSTVQLIT QLMPFGCLLD
801 YVREHKDNIG SQYLLNWCVQ IAKGMNYLED RRLVHRDLAA RNVLVKTPQH
851 VKITDFGLAK LLGAEEKEYH AEGGKVPIKW MALESILHRI YTHQSDVWSY
901 GVTVWELMTF GSKPYDGIPA SEISSILEKG ERLPQPPICT IDVYMIMVKC
951 WMIDADSRPK FRELIIEFSK MARDPQRYLV IQGDERMHLP SPTDSNFYRA
1001 LMDEEDMDDV VDADEYLIPQ QGFFSSPSTS RTPLLSSLSA TSNNSTVACI
1051 DRNGLQSCPI KEDSFLQRYS SDPTGALTED SIDDTFLPVP EYINQSVPKR
1101 PAGSVQNPVY HNQPLNPAPS RDPHYQDPHS TAVGNPEYLN TVQPTCVNST
1151 FDSPAHWAQK GSHQISLDNP DYQQDFFPKE AKPNGIFKGS TAENAEYLRV
1201 APQSSEFIGA

ERBB2_HUMAN|P04626

Coverage: 569/1255 (45%)

1 MELAALCRWG LLLALLPPGA ASTQVCTGTD MKLRLPASPE THLDMLRHLY
51 QGCQVVQGNL ELTYLPTNAS LSFLQDIQEV QGYVLIAHNQ VRQVPLQRLR
101 IVRGTQLFED NYALAVLDNG DPLNNTTPVT GASPGGLREL QLRSLTEILK
151 GGVLIQRNPQ LCYQDTILWK DIFHKNNQLA LTLIDTNRSR ACHPCSPMCK
201 GSRCWGESSE DCQSLTRTVC AGGCARCKGP LPTDCCHEQC AAGCTGPKHS
251 DCLACLHFNH SGICELHCPA LVTYNTDTFE SMPNPEGRYT FGASCVTACP
301 YNYLSTDVGS CTLVCPLHNQ EVTAEDGTQR CEKCSKPCAR VCYGLGMEHL
351 REVRAVTSAN IQEFAGCKKI FGSLAFLPES FDGDPASNTA PLQPEQLQVF
401 ETLEEITGYL YISAWPDSLP DLSVFQNLQV IRGRILHNGA YSLTLQGLGI
451 SWLGLRSLRE LGSGLALIHH NTHLCFVHTV PWDQLFRNPH QALLHTANRP
501 EDECVGEGLA CHQLCARGHC WGPGPTQCVN CSQFLRGQEC VEECRVLQGL
551 PREYVNARHC LPCHPECQPQ NGSVTCFGPE ADQCVACAHY KDPPFCVARC
601 PSGVKPDLSY MPIWKFPDEE GACQPCPINC THSCVDLDDK GCPAEQRASP
651 LTSIISAVVG ILLVVVLGVV FGILIKRRQQ KIRKYTMRRL LQETELVEPL
701 TPSGAMPNQA QMRILKETEL RKVKVLGSGA FGTVYKGIWI PDGENVKIPV
751 AIKVLRENTS PKANKEILDE AYVMAGVGSP YVSRLLGICL TSTVQLVTQL
801 MPYGCLLDHV RENRGRLGSQ DLLNWCMQIA KGMSYLEDVR LVHRDLAARN
851 VLVKSPNHVK ITDFGLARLL DIDETEYHAD GGKVPIKWMA LESILRRRFT
901 HQSDVWSYGV TVWELMTFGA KPYDGIPARE IPDLLEKGER LPQPPICTID
951 VYMIMVKCWM IDSECRPRFR ELVSEFSRMA RDPQRFVVIQ NEDLGPASPL
1001 DSTFYRSLLE DDDMGDLVDA EEYLVPQQGF FCPDPAPGAG GMVHHRHRSS
1051 STRSGGGDLT LGLEPSEEEA PRSPLAPSEG AGSDVFDGDL GMGAAKGLQS
1101 LPTHDPSPLQ RYSEDPTVPL PSETDGYVAP LTCSPQPEYV NQPDVRPQPP
1151 SPREGPLPAA RPAGATLERP KTLSPGKNGV VKDVFAFGGA VENPEYLTPQ
1201 GGAAPQPHPP PAFSPAFDNL YYWDQDPPER GAPPSTFKGT PTAENPEYLG
1251 LDVPV

PHLA1_HUMAN|Q8WV24

Coverage: 28/401 (7%)

1 MRRAPAAERL LELGFPPRCG RQEPPFPLGV TRGWGRWPIQ KRREGARPVP
51 FSERSQEDGR GPAARSSGTL WRIRTRLSLC RDPEPPPPLC LLRVSLLCAL
101 RAGGRGSRWG EDGARLLLLP PARAAGNGEA EPSGGPSYAG RMLESSGCKA
151 LKEGVLEKRS DGLLQLWKKK CCILTEEGLL LIPPKQLQHQ QQQQQQQQQQ
201 QQQQPGQGPA EPSQPSGPAV ASLEPPVKLK ELHFSNMKTV DCVERKGKYM
251 YFTVVMAEGK EIDFRCPQDQ GWNAEITLQM VQYKNRQAIL AVKSTRQKQQ
301 HLVQQQPPSQ PQPQPQLQPQ PQPQPQPQPQ PQSQPQPQPQ PKPQPQQLHP
351 YPHPHPHPHS HPHSHPHPHP HPHPHQIPHP HPQPHSQPHG HRLLRSTSNS
401 A

PHLA2_HUMAN|Q53GA4

Coverage: 15/152 (10%)

1 MKSPDEVLRE GELEKRSDSL FQLWKKKRGV LTSDRLSLFP ASPRARPKEL
51 RFHSILKVDC VERTGKYVYF TIVTTDHKEI DFRCAGESCW NAAIALALID
101 FQNRRALQDF RSRQERTAPA APAEDAVAAA AAAPSEPSEP SRPSPQPKPR
151 TP

Key Minimum PSMs: 0 1 2 5 Modified

Supplementary Figure 11. Peptide coverage report for mass spectrometry results.

Protein sequence coverage maps of selected proteins in the 100 nM TAK-285 global protein profiling data set. Identified PSMs are represented in color based on their occurrence shown in the figure key.

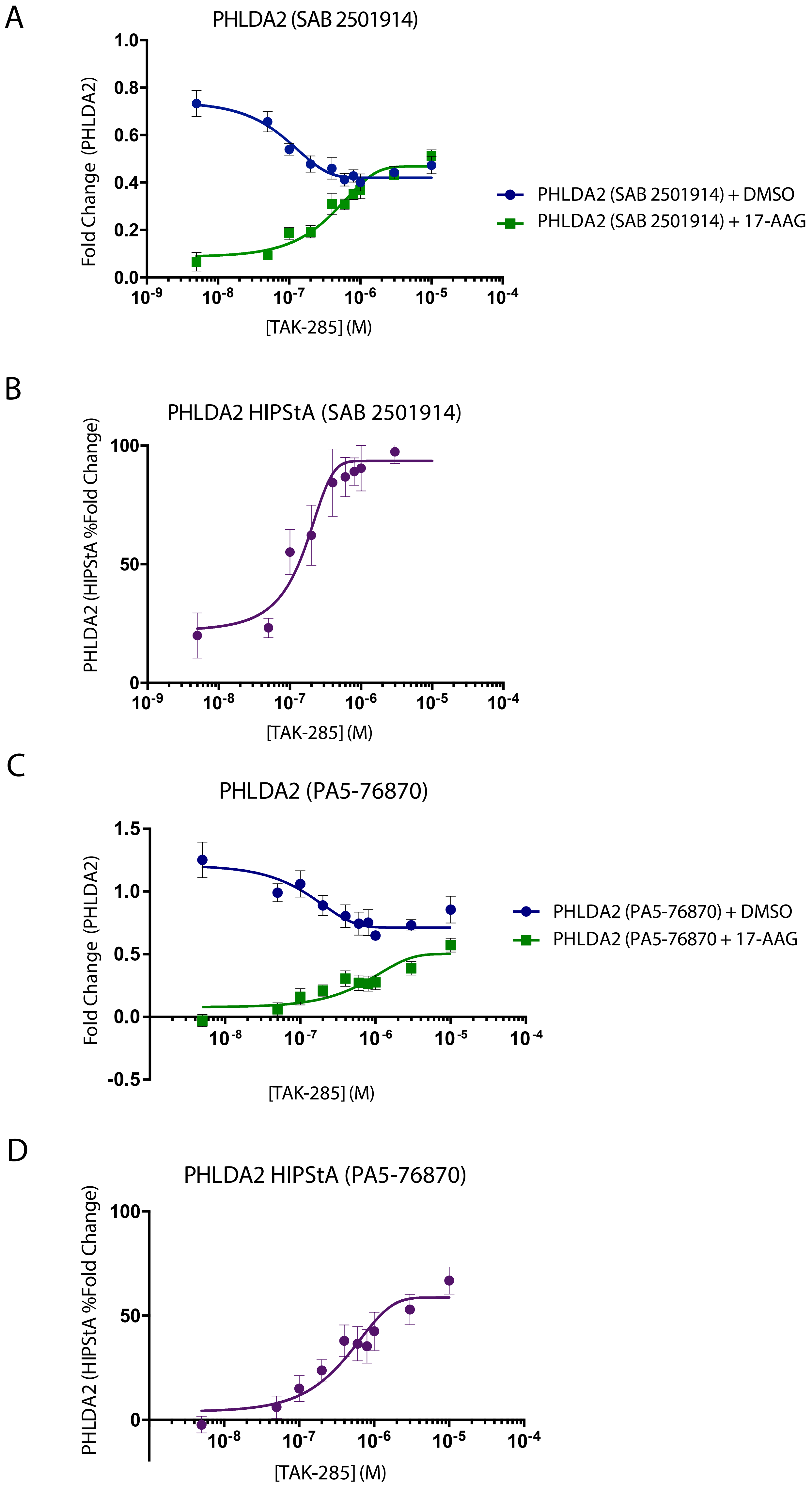

A
PHLDA2 (SAB 2501914)
Fold Change (PHLDA2)
[TAK-285] (M)
PHLDA2 (SAB 2501914) + DMSO
PHLDA2 (SAB 2501914) + 17-AAG
B
PHLDA2 HIPStA (SAB 2501914)
PHLDA2 (HIPStA %Fold Change)
[TAK-285] (M)
C
PHLDA2 (PA5-76870)
Fold Change (PHLDA2)
[TAK-285] (M)
PHLDA2 (PA5-76870) + DMSO
PHLDA2 (PA5-76870 + 17-AAG
D
PHLDA2 HIPStA (PA5-76870)
PHLDA2 (HIPStA %Fold Change)
[TAK-285] (M)

Supplementary Figure 12. Validation of PHLDA2 as a TAK-285 target using HIPStA quantitative immunofluorescence imaging.

A) Measurement of the relative PHLDA2 protein levels in MCF-7 neoHER2 cells pre-treated with a titration of TAK-285 then treated with 1 μM 17-AAG. PHLDA2 protein levels are expressed as the fraction of PHLDA2 present in DMSO treated samples detected using antibody SAB 2501914. B) Summarized PHLDA2 HIPStA %fold change calculated relative to PHLDA2 levels for each individual concentration of TAK-285 in (A) The HIPStA EC_{50} for TAK-285 association with PHLDA2 in this study was 119 nM . C) Measurement of the relative PHLDA2 protein levels in MCF-7 neoHER2 cells pre-treated with a titration of TAK-285 then treated with 1 μM 17-AAG. PHLDA2 protein levels are expressed as the fraction of PHLDA2 present in DMSO treated samples detected using antibody PA5-76870. The HIPStA EC_{50} for TAK-285 association with PHLDA2 was 481 nM. D) Summarized PHLDA2 HIPStA %fold change calculated relative to PHLDA2 levels for each individual concentration of TAK-285 in (C). Data are represented as mean values with error bars representing standard error of mean (n=8). SAB 2501914 (Sigma) was raised against the peptide C-EPSRPSPQPKPRTP (C-terminal region of PHLDA2), while PA5-76870 was raised against full length recombinant human PHLDA2. Source data are provided as a Source Data file.

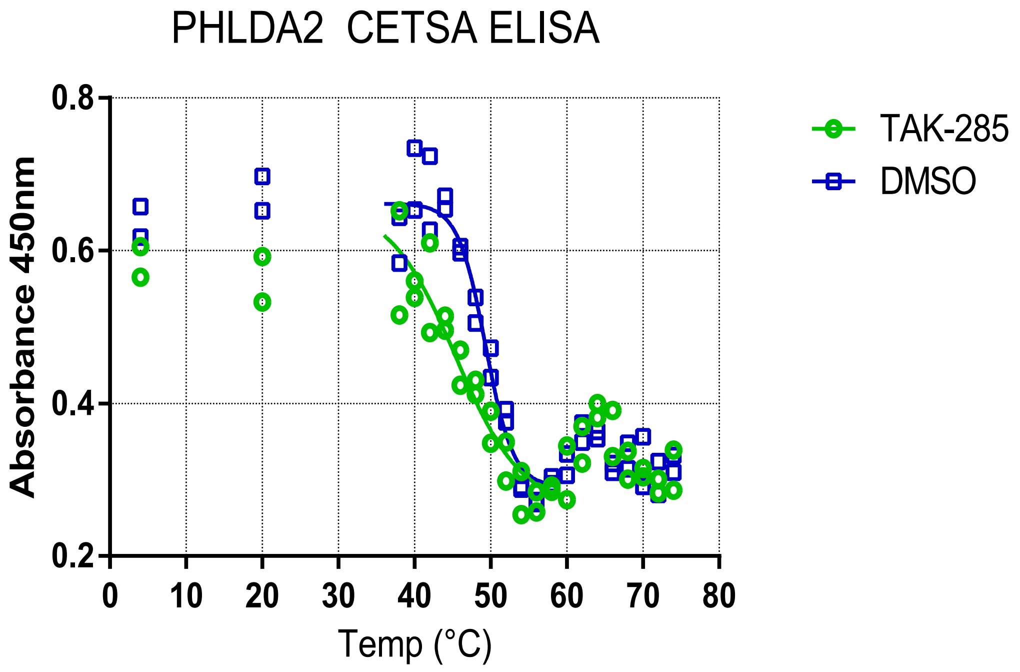

Supplementary Figure 13. CETSA study of the effect of TAK-285 on the thermal stability of PHLDA2 in cell lysate.

MCF7 neoHER2 cell lysate was aliquoted into duplicate samples, which were pre-incubated (1 hour, 4º C) with either 10 µM TAK-285, or DMSO control, and then were heated to defined temperatures indicated on the x-axis. Precipitated protein was removed by centrifugation and the PHLDA2 protein remaining in the supernatant was measured using a PHLDA2 sandwich enzyme linked immunosorbent assay (ELISA) by measuring absorbance at 450 nm. Data represent individual data points from biological duplicates for each temperature. A four parameter curve fit (using PRISM) was applied to data between the temperature range 36 to 58ºC. The presence of TAK-285 caused a >4ºC decrease in the thermal denaturation of PHLDA2, indicating a destabilization of PHLDA2 by TAK-285. Data are representative of two independent experiments. Source data are provided as a Source Data file.

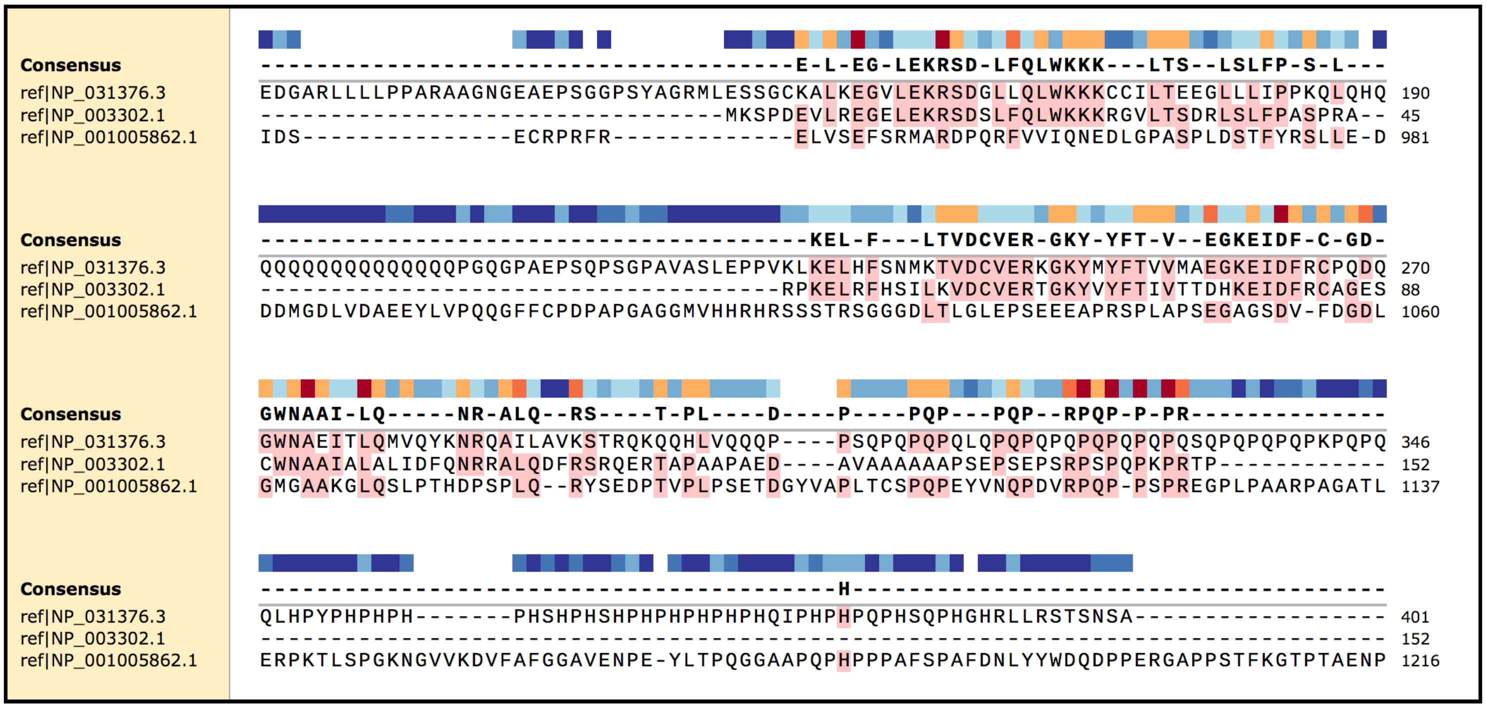

Supplementary Figure 14. Sequence alignment of HER2 vs. PHLDA1 and PHLDA2.

Sequence alignments using NCBI COBALT, based on conserved domain and local sequence similarity. Reference sequences: NP_031376.3 (PHLDA1), NP_003302.1 (PHLDA2), NP_001005862 (ERBB2). The region of HER2 with the greatest homology to PHLDA1 and PHLDA2 is within the kinase domain of HER2.

Drug	HER2 IC50 (nM)
Lapatinib	25.4
HY-14674	18.5
TAK-285	42.7

Supplementary Table 1. Biochemical IC50 values for the inhibition of HER2.

Empirically determined biochemical IC50 values for the inhibition of HER2. Kinase assays were performed using the ZLYTE assay format using ATP concentrations defined at the ATP K_M value for HER2.
