# Supplementary material for: Chaperone mediated detection of small molecule target binding in cells
Source: Nat Commun. 2020 Jan 23;11:465. doi: 10.1038/s41467-019-14033-0 (PMC6978363; doi:10.1038/s41467-019-14033-0)
Supplement: Supplementary file 3 — Description of Additional Supplementary Files [file 41467_2019_14033_MOESM3_ESM.docx]

File Name: Supplementary Data 1

Description: Biochemical kinase selectivity profiling of TAK-285 across a panel of 235 protein kinase biochemical assays. Data show the percentage inhibition of each kinase tested using the ZLYTE assay format and ATP concentration of 10μM.

File Name: Supplementary Data 2

Description: A comparison of HIPStA and CETSA data for those proteins with a significant response in the HIPStA global proteomics profiling study. The table compares the HIPStA and published CETSA data (Savitski et al 2014) for proteins that demonstrated both a significant reduction by 17-AAG and a significant elevation by pretreatment with staurosporine relative to the 17-AAG only samples in the HIPStA study. Statistical significance was determined using a student t-test with two-tailed comparison assuming equal variance and p<0.05. The tabulated data from Savitski et al 2014 were combined with the HIPStA global proteomics profiling data depicted in Supplemental Figure 8 using gene names to match the data sets. Only data for fully annotated genes were used for the data comparison. Relative reduction in protein levels greater than 15% (log2ratio < -0.235) are highlighted in blue. Relative elevation in protein levels greater than 15% (log2ratio > 0.202) are highlighted in red.

File Name: Supplementary Data 3.

Description: A Comparison of HIPStA and CETSA data for those proteins that were defined as having a significant interaction with staurosporine in the published CETSA study (Savitski et al 2014). The tabulated data from Savitski et al were combined with the HIPStA global proteomics profiling data depicted in Supplemental Figure 8 using gene names to match the two data sets. Only data for fully annotated genes were used for the data comparison. HIPStA relative reduction in protein levels greater than 15% (log2ratio < -0.235) are highlighted in blue. Relative elevation in protein levels greater than 15% (log2ratio > 0.202) are highlighted in red. Statistical significance is highlighted in green (statistical significance was determined using a student t-test with two-tailed comparison assuming equal variance and p<0.05).
