# Supplementary material for: Chaperone mediated detection of small molecule target binding in cells
Source: Nat Commun. 2020 Jan 23;11:465. doi: 10.1038/s41467-019-14033-0 (PMC6978363; doi:10.1038/s41467-019-14033-0)
Supplement: Supplementary file 7 — Reporting Summary [file 41467_2019_14033_MOESM7_ESM.pdf]

nature research

Corresponding author(s): Robert Blake

Last updated by author(s): Dec 4, 2019

Reporting Summary

Nature Research wishes to improve the reproducibility of the work that we publish. This form provides structure for consistency and transparency in reporting. For further information on Nature Research policies, see Authors & Referees and the Editorial Policy Checklist .

Statistics

For all statistical analyses, confirm that the following items are present in the figure legend, table legend, main text, or Methods section.

n/a	Confirmed	
☐	☒	The exact sample size (*n*) for each experimental group/condition, given as a discrete number and unit of measurement
☐	☒	A statement on whether measurements were taken from distinct samples or whether the same sample was measured repeatedly
☐	☒	The statistical test(s) used AND whether they are one- or two-sided<br>*Only common tests should be described solely by name; describe more complex techniques in the Methods section.*
☐	☒	A description of all covariates tested
☐	☒	A description of any assumptions or corrections, such as tests of normality and adjustment for multiple comparisons
☐	☒	A full description of the statistical parameters including central tendency (e.g. means) or other basic estimates (e.g. regression coefficient) AND variation (e.g. standard deviation) or associated estimates of uncertainty (e.g. confidence intervals)
☐	☒	For null hypothesis testing, the test statistic (e.g. *F*, *t*, *r*) with confidence intervals, effect sizes, degrees of freedom and *P* value noted<br>*Give P values as exact values whenever suitable.*
☒	☐	For Bayesian analysis, information on the choice of priors and Markov chain Monte Carlo settings
☒	☐	For hierarchical and complex designs, identification of the appropriate level for tests and full reporting of outcomes
☒	☐	Estimates of effect sizes (e.g. Cohen's *d*, Pearson's *r*), indicating how they were calculated

Our web collection on statistics for biologists contains articles on many of the points above.

Software and code

Policy information about availability of computer code

Data collection	TMT reporter ions produced by the TMT tags were quantified with an in-house software package known as Mojave. Protein abundance ratios were computed in R (3.2.2).
Data analysis	MS/MS spectra were searched using Mascot (v.2.4.1) against UniProt DB (2015_04). Gene Ontology enrichment analysis was performed using the 'hyperGTest' function in the R package 'GOstats' (version 2.48.0). Quantitative fluorescence image analysis was carried out using HCS Navigator Version 6.6.0 (Build 8153) Classic Scan software associated with a CellInsight CX7 High-Content Screening (HCS) Platform (Thermo Fisher Scientific).

For manuscripts utilizing custom algorithms or software that are central to the research but not yet described in published literature, software must be made available to editors/reviewers. We strongly encourage code deposition in a community repository (e.g. GitHub). See the Nature Research guidelines for submitting code & software for further information.

Data

Policy information about availability of data

All manuscripts must include a data availability statement. This statement should provide the following information, where applicable:

- Accession codes, unique identifiers, or web links for publicly available datasets
- A list of figures that have associated raw data
- A description of any restrictions on data availability

All mass spectrometry raw files have been deposited into the MassIVE database (http://massive.ucsd.edu/) and can be downloaded by the identifier MSV000084586 as well as in ProteomeXchange with accession number PXD016301.
Figures 1, 2, 3 and 4 as well as Supplementary Figures 1 to 14 have associated raw data in a Source Data File ("Source Data").
Supplementary figures 8, 9 and 10 have associated raw data in 2 and 3.

Field-specific reporting

Please select the one below that is the best fit for your research. If you are not sure, read the appropriate sections before making your selection.

☒ Life sciences ☐ Behavioural & social sciences ☐ Ecological, evolutionary & environmental sciences

For a reference copy of the document with all sections, see nature.com/documents/nr-reporting-summary-flat.pdf

Life sciences study design

All studies must disclose on these points even when the disclosure is negative.

Sample size	No sample size calculation was performed. Sample sizes were chosen based on practical limitations of experimental design and data collection
Data exclusions	No data exclusions were applied
Replication	All quantitative fluorescence imaging experiments are representative of 3 or more similar experiments. Mass spectrometry-based global protein profiling was carried out on 2 completely independent sample sets. Where proteins are highlighted as having significant changes the observations were consistent between all data sets.
Randomization	Randomization of samples was not relevant to the studies
Blinding	Blinding was not relevant to the studies

Reporting for specific materials, systems and methods

We require information from authors about some types of materials, experimental systems and methods used in many studies. Here, indicate whether each material, system or method listed is relevant to your study. If you are not sure if a list item applies to your research, read the appropriate section before selecting a response.

Materials & experimental systems

n/a	Involved in the study	
☐	☒	Antibodies
☐	☒	Eukaryotic cell lines
☒	☐	Palaeontology
☒	☐	Animals and other organisms
☒	☐	Human research participants
☒	☐	Clinical data

Methods

n/a	Involved in the study	
☒	☐	ChIP-seq
☒	☐	Flow cytometry
☒	☐	MRI-based neuroimaging

Antibodies

Antibodies used

ERalpha antibody, Santa Cruz Biotech, sc-8002 (ERα (F-10), lot number C1213, is a mouse monoclonal antibody specific for an epitope between amino acids 570-595 at the C-terminus of human ERα.

HER2 antibody, abcam, ab134182 (Rabbit monoclonal [EP1045Y] to ErbB2), lot number GR149911-22, recognizes an epitope within Human ErbB2 between aa 1200 and the C-terminus and detects unphosphorylated ErbB2 as well as ErbB2 phosphorylated at Tyr1248.

CRAF antibody, abcam, ab50858 (mouse monoclonal [RNP1] to Raf1 (CRAF GQRDSSYYWEIE), lot number GR170300-19, recognizes an epitope corresponding to amino acids 334-345 of Human CRAF (Raf1).

Two independent antibodies were used to confirm the effects of TAK-285 and 17-AAG on PHLDA2 protein: Sigma Aldrich, SAB 2501914 lot number 6184P1 (Goat polyclonal antibody raised to the peptide with sequence C-EPSRPSPQPKPRTP, from the C-terminal region of the protein sequence according to NP_003302.1); and rabbit polyclonal antibody raised to full-length PHLDA2 (ThermoFisher, PA5-76870, Lot Number TG2605843; the antibody was affinity-purified from rabbit antiserum by affinity-chromatography using epitope-specific immunogen).

The secondary antibodies used for quantitative fluorescence imaging were: Alexafluor 488 conjugate anti-mouse IgG Life Technologies #A1202; Alexafluor 488 conjugate anti-rabbit IgG, Life Technologies #A1206; Alexafluor 488 conjugate anti-goat Life Technologies # A-11055.

Validation

Specificity of Santa Cruz Biotech, sc-8002 (ERα (F-10) was confirmed using the selective estrogen receptor degrader (SERD) effect of fulvestrant to decrease the immunofluorescence signal by 90%.

The immunofluorescence staining pattern of abcam ab134182 (Rabbit monoclonal [EP1045Y] to HER2 (ErbB2) was consistent with a plasma membrane localization. The specificity of the effects of TAK-285 and 17-AAG on HER2 protein levels were independently confirmed by mass-spec based proteomics analysis.

The specificity of CRAF antibody, abcam, ab50858 (mouse monoclonal [RNP1] to CRAF was confirmed in an experiment in which doxycyclin induced expression of an shRNA to CRAF induced a 30% decrease in signal as measured by quantitative fluorescence

imaging.
The specificities of the PHLDA2 antibodies were confirmed by using two independent antibodies to observe the same effects (TAK-285 and 17-AAG modulate PHLDA2 protein levels) and the observations were corroborated using the orthogonal techniques of quantitative fluorescence imaging and mass-spec based proteomics analysis.

Eukaryotic cell lines

Policy information about cell lines

Cell line source(s)	The human breast adenocarcinoma cell line MCF7 neo/HER2 and human lung carcinoma cell line A549 were originally sourced from Genentech's cell line repository gCell.
Authentication	The A549 lung carcinoma (ATCC® CCL-185), SK-BR-3 breast adenocarcinoma (ATCC® HTB-30) and AU565 breast adenocarcinoma (ATCC® CRL-2351) cell lines were obtained from Genentech's cell line repository (gCell) and originated from ATCC. The cancer cell line MCF-7-neoHER2 was obtained from Genentech's cell line repository (gCell) and is an in vivo-selected line that expresses high levels of HER2 (3+) driven from an expression plasmid carrying a neomycin resistance gene and has the activating E545K PI3K mutation present in the parental MCF-7 cell line (ATCC® HTB-22).<br>The identity of the MCF-7-neoHER2, A549, SK-BR-3 and AU565 cell identity was confirmed by genotyping QC by Genentech cell line repository (gCell).
Mycoplasma contamination	Cell lines were tested for mycoplasma contamination and shown to be negative by Genentech cell line repository (gCell).
Commonly misidentified lines<br>(See ICLAC register)	*Name any commonly misidentified cell lines used in the study and provide a rationale for their use.*
